# The Mobilization of Aluminum into the Biosphere

**DOI:** 10.3389/fneur.2014.00262

**Published:** 2014-12-08

**Authors:** Aileen I. Pogue, Walter J. Lukiw

**Affiliations:** ^1^Alchem Biotech, Toronto, ON, Canada; ^2^Louisiana State University Neuroscience Center and Department of Ophthalmology, Louisiana State University School of Medicine, New Orleans, LA, USA; ^3^Department of Neurology, Louisiana State University Health Sciences Center, New Orleans, LA, USA

**Keywords:** aluminum, biosphere, passivation layer, Hall–Heroult–Bayer, ecosystems

Aluminum is currently the most widely used non-ferrous metal, and its extraction and purification from geological stores exceeds that of any other metal except iron (1, 2). In 2013, global primary aluminum production was ~52 million tons (104 billion pounds) or about 15 pounds for very person on the earth (1–4). The global outlook for aluminum demand from developing countries such as Brazil, China, India, and Indonesia is rapidly increasing, due to new applications for aluminum and aluminum alloys in infrastructural support, transportation including automobiles, aviation and aerospace applications, electrical transmission, and the generation of energy, including catalytic *zeolites* in the petroleum and petrochemical industries (5). Interestingly, the largest “*machine*” built by humankind is the domestic and international networks for the transmission of electricity. Although traditionally-used copper has a higher electrical conductivity, aluminum is only slightly less so, being lighter, more ductile, and less expensive; aluminum is now widely used for *both* high-voltage tower construction and the electrical transmission wires themselves (2–5). It has been estimated that within the next 10 years aluminum production will exceed that of the previous 150 years (1–3). *This prolific de novo generation of aluminum combined with its highly efficient recycling means this metal is becoming increasingly present in our biosphere, defined as the sum of all ecosystems and living organisms on the earth*. This short “Opinion” paper will overview and comment on the current massive mobilization of aluminum into the earth’s biosphere.

## Aluminum Geology, Historical and Industrial Perspectives

Bound tightly by oxygen and silicon, aluminum oxides and silicates, commonly referred to as alumina and/or aluminosilicate, exist naturally in ores generically termed *bauxite*. Bauxite consists mainly of the hydrated aluminum oxide (Al_2_O_3_xH_2_O) minerals *gibbsite*, *boehmite*, and *diaspore*, and is the world’s main source of raw material for the production of aluminum. Aluminum is extremely abundant; and after oxygen and silicon is the third most abundant element in the earth’s crust and the most abundant metal (1–6). Bauxite ore often contains several varieties of iron oxides, mostly *goethite*, *hematite*, and the clay mineral *kaolinite* making them reddish in appearance; widely distributed in the tropics, rusty-red soil types called *laterites* are highly enriched in complex aluminum- and iron-oxides. Interestingly, as the primordial earth cooled, the lighter, lowest-density elements rose to the surface crust, and aluminum, one of the lightest metals known, currently exists in relatively easy-to-access near-the-surface deposits (7). Hence, two geophysical features make bauxite relatively easy to acquire as (i) massive bauxite deposits lie very near the earth’s surface, conducive to strip mining, with little or no overburden to remove; and (ii) the aluminum content of bauxite is very high in the lithosphere, conducive to vast bauxite mining and smelting operations. It is not often appreciated that although aluminum *averages* 8% (w/v) of the entire earth’s crust, alumina-enriched bauxite ore deposits can often reach up to 50% (w/v); for example, the Gove and Weipa bauxite deposits of Northern Territory and Queensland, Australia contain ~50% available alumina, and are currently among the largest, most accessible, and highest grade bauxite mines in the world (4, 6). Remarkably, the largest aluminum mines in Australia can extract ~3,000 tons (6,000,000 pounds) of bauxite per hour, and these are the largest contributors to a global aluminum production, which is currently in excess of about ~6,000 tons (12 million pounds) of 99% pure aluminum *produced every hour of every day* (2–5). The virtual inexhaustible supply of alumina in the earth’s crust combined with the high recycling potential for aluminum (see below) guarantee to make aluminum an expanding presence and permanent fixture in our biosphere for the foreseeable future.

Alumina, aluminosilicates, and bauxite are relatively inert, naturally occurring compounds, in contrast to aluminum’s extremely high reactivity in its pure elemental form (3). Aluminum was first produced experimentally in 1825 by the Danish chemist Hans Christian Oersted, and later the German, French, and Austrian chemists Friedrich Wöhler, Henri Sainte-Claire Deville, and Carl Joseph Bayer up-graded isolation efficiencies and purification technologies, (7, 8). Just ~75 years later, the inception and application of the Hall–Heroult–Bayer process, and later modifications and upgrades of this industrial technology, including implementation of the Soderberg and prebake technologies, has made aluminum mining, extraction, and purification a multibillion international industry. Global aluminum production since 1900 has increased an amazing ~13,000-fold (3, 5, 7). In the currently used Hall–Heroult–Bayer process molten cryolite (Na_3_AlF_6_) is used as a solvent for alumina (Al_2_O_3_) and subsequent energy-intensive direct current electrolysis refines the aluminum (melting point ~660°C; ~1221°F) to >99% purity in a single electrochemical step (7–10). The most important chemical reaction in this operation is: 2(Al_2_O_3_) + electricity − >4Al^3+^ + 3O_2_; it takes about 2 tons of alumina and 17000 kWh of electricity to produce 1 ton of pure aluminum (5, 7). Interestingly, the formidable amount of energy required to produce aluminum has prompted the Russian giant metal company Rusal, now the world’s largest supplier of aluminum (with a 12% global share of the aluminum market) to construct the world’s first nuclear powered aluminum smelter in the Saratov region of southern Russia with a production capacity of ~2.3 million tons per year (~525,000 pounds per hour) (10). Recycling aluminum requires only about 5% of the original production input energy, and yearly about 20 million tons (40 billion pounds) of aluminum are recycled; for example, in some Scandinavian countries yearly over 90% of aluminum is recycled (6). Remarkably, it has been estimated that *approximately two-thirds of all aluminum ever produced since 1900 is still in use*, partly because it is so easy to recycle it into a form that has properties virtually identical to those of “virgin” aluminum generated by primary aluminum smelting operations, (1–5, 8, 9).

## Aluminum Bonding to Oxygen Donor Ligands and Passivation

The geochemistry of aluminum is relatively simple when it occurs naturally, but becomes considerably more complex when it enters our biosphere and into the biology of living organisms. The unchanging 3^+^ valence of aluminum and small ionic radius of 0.5 nm make it an unusually high-charge density species with *Z*^2^/*r* = 18 (where *Z* = ionic charge and *r* = ionic radius); in fact, aluminum has by far the highest charge density of any biosphere-abundant element (7, 8, 11). Except in biological and/or environmentally acidic situations, aluminum remains tightly bound to oxygen in geological stores. For example, the metal–oxygen (Me–O) bond dissociation energy, an indicator of how strong chemical bonds are, and how much energy is required to break them, is 122 kcal/mol for Al–O and 98 kcal/mol for Fe–O; for comparison, the Al–Al and Fe–Fe dissociation energies are significantly less at 44 and 24 kcal/mol, respectively (7–9). This indicates that the Al–O bond is exceedingly strong and that Al has a higher affinity for O than does Fe for O, Fe for Fe, or Al for Al (in comparison the Si–O and Si–Si bond energies are 191 and 78 kcal/mol, respectively) (11–14). The strong Al–O bonding is responsible for the extremely high resistance of metallic aluminum to weathering and the decomposition of aluminum through contact and interaction with the earth’s biosphere (see below). On the other hand, pure aluminum is extremely reactive with atmospheric oxygen, and a thin, highly protective “*passivation layer*” of aluminum oxide up to ~4 nm in thickness (8 times the radius of the Al^3+^ ion) rapidly forms on exposed aluminum surfaces thus creating a physical barrier to corrosion that prevents further oxidation (7, 11, 14). The chemistry of aluminum in geological stores is very strongly related to the capacity of aluminum to form an Al–O “passivation layer”; however, this reactivity situation changes when aluminum is exposed to the complex mixtures of oxygen donor ligands and physiological conditions normally abundant in living organisms, which populate the biosphere.

## Aluminum, the Biosphere and Biology

The biosphere, sometimes defined as “*the self-regulating zone of all life on earth*,” and further divided into (i) the atmosphere that except under extraordinary dusty conditions normally contains very little free aluminum; (ii) the lithosphere that contains all raw aluminum in geologic deposits; and (iii) the hydrosphere that includes all water bodies on the earth. The estimated crustal (lithospheric) abundance of aluminum is an amazing 82.3 g/kg while the estimated oceanic (hydrospheric) abundance of aluminum is about 2 × 10^−3^ mg/l (7, 9–11). Free Al^3+^ concentrations in land (lithospheric) based organisms are ~10^−11^ mol/l but may be compartmentalized at higher concentrations (15–17). There seems to be at least two possible explanations for these generally low Al^3+^ concentrations: either (i) the Al^3+^ locked in the earth’s crust has been too inert and inaccessible to the biochemistry of life or (ii) biological systems have evolved to reject Al^3+^ (11–13). Interestingly, in the circulating physiological fluids of biological systems, including the blood, lymph, cerebrospinal fluid, and extracellular fluids whose composition, temperature, or pH is in constant flux between various physiological tissues and compartments, Al^3+^ associates with oxygen donor ligands to counter aluminum’s 3^+^ charge (11–15). In biological systems, oxygen donor ligands typically include carboxylates, organic and inorganic phosphates, nucleotides, and polynucleotides such as DNA and RNA in all of their structural forms (11, 15–19). Interestingly, unless carboxylate groups are arranged to make strong chelation possible Al^3+^ prefers to bind to phosphates, so the millimolar concentrations of polyphosphate in the genetic material of the nucleus may be particularly attracted to aluminum (7, 10–12, 14, 20). Al^3+^ is notorious for permanently displacing normal biological metal ions, for example, Al^3+^ binds almost 10^7^ times more strongly to ATP than does Mg^2+^, the normal ATP metal ion ligand, and once Al^3+^ acquires an energetically favorable electron-rich binding site that shields its charge it has a tendency to remain there, with high refractivity to a wide variety of chelation methods (11, 15–19). To this end, while it has often been stated that there is “*no normal biological function for aluminum*,” its remarkable capacity to bind to DNA phosphates and aggregate chromatin and nucleic acids into highly compacted “heterochromatic” forms may have been used by evolution to shut down the expression of specific genetic information in selected cell types (11, 15–21). This is of note neuropathologically since only about ~1 billionth of the 15 pounds of aluminum produced yearly per person has been shown to dramatically down-regulate the expression of genes in the human brain and in doing so contribute to neurological dysfunction in a biologically detrimental disease-driving process termed “genotoxicity” (17–22). *A great need currently exists to elucidate more clearly how aluminum behaves under constantly changing physiological conditions in certain compartmentalized regions of the human body, such as in the cytoplasm and nucleus, the central nervous system and cerebrovascular circulation, and how it impacts normal immune, neurological and related biological systems*. Several excellent and extremely comprehensive reviews on the detrimental impact of aluminum on human biology have recently appeared in the scientific literature and are highly suggested reading for interested researchers of aluminum toxicity, and aluminum’s current massive integration into our biosphere (23–30).

## Concluding Remarks

The geological extraction, smelting, production, and purification of aluminum and its mobilization into the biosphere are increasing exponentially. There are no new materials with properties similar to aluminum currently available, and new applications for aluminum use and demand continue to rise. Approximately 1.1 billion tons (2.2 × 10^15^ pounds) of metallic aluminum have been extracted from geological deposits and exported into the biosphere since aluminum production began in earnest in 1900, and remarkably, through recycling, re-use and intrinsic longevity factors, about two-thirds of this amount is still in productive use (4–6). The continuing increase in the mobilization of aluminum into our biosphere is driven by at least six interdependent factors: (i) worldwide, the demand for aluminum is currently strong and continues to rise; (ii) there is virtually a limitless supply of the relatively inexpensive raw material bauxite in vast geologic stores to generate new aluminum; (iii) the shift from relatively inert aluminum oxide in bauxite in earthbound stores into metallic aluminum is a relatively straightforward, one-step, electro-thermal process constantly being streamlined and up-graded to higher efficiency technologies; (iv) the unique geochemical, biophysical, and chemical properties of aluminum make it relatively easy to mine, extract, purify, and recycle; (v) upwards of 90% of aluminum can be recycled back to into potentially bioavailable aluminum that is not cast back into inert geological stores; and (vi) once purified aluminum enters the biosphere, it remains there to be used in multiple products to which human beings have exposure.

Indeed, once “*unlocked*” from geological stores where it is relatively inactive, in many respects aluminum has become “*artificially and permanently integrated into the biosphere*” as its primary production and bioavailability have paralleled the growth of human civilization (1–9). However, along with the ongoing mobilization of aluminum into our biosphere is an expanding list of aluminum’s adverse effects on human health and welfare. Currently, the Medline database at the US National Institutes of Health (www.ncbi.nlm.nih.gov; using the keywords “aluminum” and “disease”) lists ~4100 peer-reviewed scientific papers describing multiple aspects of aluminum toxicity, and its potential contribution to a remarkably diverse number of human physiological dysfunctions and exposure-related diseases. *As can be gleaned from the many research papers in this special volume of Frontiers, humankind must be wise to temper the widespread integration of this very useful metal into the biosphere with the realization that very minute amounts of aluminum in the wrong place at the wrong time in human development, physiology and neurobiology can very often be hazardous enough to generate some serious healthcare concern*.

## Conflict of Interest Statement

The authors declare that the research was conducted in the absence of any commercial or financial relationships that could be construed as a potential conflict of interest.
